# Evaluation of angiotensin converting enzyme 2 (ACE2), angiotensin II (Ang II), miR-141-3p, and miR-421 levels in SARS-CoV-2 patients: a case-control study

**DOI:** 10.1186/s12879-024-09310-3

**Published:** 2024-04-22

**Authors:** Ehsan Kakavandi, Kaveh Sadeghi, Mohammad Shayestehpour, Hossein Mirhendi, Abbas Rahimi Foroushani, Talat Mokhtari-Azad, Nazanin Zahra Shafiei Jandaghi, Jila Yavarian

**Affiliations:** 1 Department of Virology, School of Public Health, Tehran University of Medical Sciences, Tehran, Iran; 2 Department of Bacteriology and Virology, Faculty of Medicine, Isfahan University of Medical Sciences, Isfahan, Iran; 3 Department of Medical Parasitology and Mycology, School of Medicine, Isfahan University of Medical Sciences, Isfahan, Iran; 4 Department of Epidemiology and Biostatistics, School of Public Health, Tehran University of Medical Sciences, Tehran, Iran; 5 Research Center for Antibiotic Stewardship and Antimicrobial Resistance, Tehran University of Medical Sciences, Tehran, Iran

**Keywords:** Angiotensin converting enzyme 2, COVID-19, Angiotensin II, SARS-CoV-2, miR-141-3p, miR-421

## Abstract

**Background:**

Severe acute respiratory syndrome coronavirus 2 (SARS-CoV-2) is a highly contagious virus that uses angiotensin converting enzyme 2 (ACE2), a pivotal member of the renin–angiotensin system (RAS), as its cell-entry receptor. Another member of the RAS, angiotensin II (Ang II), is the major biologically active component in this system. There is growing evidence suggesting that serum miRNAs could serve as prognostic biomarkers for SARS-CoV-2 infection and regulate ACE2 expression. Therefore, the aim of this study is to evaluate the changes in the serum levels of sACE2 and Ang II, as well as the expression level of miR-141-3p and miR-421 in SARS-CoV-2 positive and negative subjects.

**Methods:**

In the present study, the serum levels of sACE2 and Ang II were measured in 94 SARS-CoV-2 positive patients and 94 SARS-CoV-2 negative subjects with some symptoms similar to those of SARS-CoV-2 positive patients using the ELISA method. In addition, the expression level of miR-141-3p and miR-421 as ACE2 regulators and biomarkers was evaluated using quantitative real-time PCR (qRT-PCR) method.

**Results:**

The mean serum sACE2 concentration in the SARS-CoV-2-positive group was 3.268 ± 0.410 ng/ml, whereas in the SARS-CoV-2 negative group, it was 3.564 ± 0.437 ng/ml. Additionally, the mean serum Ang II level in the SARS-CoV-2 positive and negative groups were 60.67 ± 6.192 ng/L and 67.97 ± 6.837 ng/L, respectively. However, there was no significant difference in the serum levels of sACE2 (*P* value: 0.516) and Ang II (*P* value: 0.134) between the SARS-CoV-2 positive and negative groups. Meanwhile, our findings indicated that the expression levels of miR-141-3p and miR-421 in SARS-CoV-2 positive group were significantly lower and higher than SARS-CoV-2 negative group, respectively (*P* value < 0.001).

**Conclusions:**

Taken together, the results of this study showed that the serum levels of sACE2 and Ang II in SARS-CoV-2 positive and negative subjects were not significantly different, but the expression levels of miR-141-3p and miR-421 were altered in SARS-CoV-2 positive patients which need more investigation to be used as biomarkers for COVID-19 diagnosis.

## Introduction

At the end of 2019, a novel coronavirus named severe acute respiratory syndrome-coronavirus-2 (SARS-CoV-2) was first officially reported in Wuhan, the capital of Hubei Province in China. However, a number of studies have indicated that the virus was already prevalent in some parts of the world prior to this report [1, 2]. SARS-CoV-2 was highly transmissible which spread all over the world, resulting in a serious pandemic. The clinical symptoms of COVID-19 (Coronavirus disease 2019) are fever, fatigue, myalgia, cough (most common), headache, sore throat, diarrhea, and loss of smell and taste (less common). A better understanding of SARS-CoV-2 pathogenesis may lead to the identification of more effective therapeutic and preventive strategies. Current studies suggest that the pathogenesis of SARS-CoV-2 pneumonia comprises of two phases. The first phase is viral replication and subsequent direct tissue damage by the replicating virus. The second phase includes immune hyperactivity (the recruitment of immune cells that result in local/systemic inflammatory responses) [3, 4].

The SARS-CoV-2 receptor is angiotensin-converting enzyme 2 (ACE2), which plays a critical role in the pathogenesis of COVID-19 [5]. ACE2 is a transmembrane glycoprotein that is located on the cell membrane (known as the membrane-bound form or mACE2), but does not always remain on the cell surface. For instance, it can be detached through an event called shedding induced by disintegrin and metalloprotease 17 (ADAM17), which produces the soluble form of ACE2 (sACE2), resulting in the loss of mACE2 [6]. ACE2 has important functions in the renin–angiotensin system (RAS): blood pressure maintenance and electrolyte homeostasis [7–9]. In the RAS, after releasing renin into the blood, it cleaves angiotensinogen to angiotensin I (Ang I), and then ACE catalyzes the conversion of Ang I to Ang II. Ang II, the major biologically active component of the RAS, has several effects, including vasoconstriction, hypertension, thrombosis, and inflammation. The final consequence of the RAS axis depends on the balance between ACE and ACE2 [10]. Additionally, previous studies have shown that there are possible correlations between RAS and the clinical outcomes/pathogenesis of acute respiratory distress syndrome (ARDS) [11–14]. The binding of the SARS-CoV-2 spike protein to the cell surface of ACE2 results in mACE2 cleavage at the enodomain and ectodomain sites. This process triggers the shedding of cellular mACE2 receptors, which results in their systemic release into the blood circulation, which is associated with the severity of COVID-19 [15, 16]. On the other hand, several studies have demonstrated that Ang II levels are correlated with the severity and mortality of respiratory virus-infected patients [17].

However, despite rapid progress in understanding of the pathophysiological roles of ACE2, little is known about the mechanisms that regulate its expression. Numerous investigations have documented that microRNAs (miRNAs) possess the ability to regulate ACE2 expression in various diseases [18–20]. miRNAs are small non-coding RNAs (with an average of 22 nucleotides) capable of negatively regulating gene expression [21]. There is growing evidence suggesting that serum miRNAs could serve as prognostic biomarkers for SARS-CoV-2 infection. Various studies have shown the regulation of ACE2 expression by different miRNAs, including miR-17-5p, miR-5197-3p, miR-212-p, miR-20b-5p, miR-4677-3p, and miR-3909 which have been demonstrated to have a direct impact on the SARS-CoV-2 genome, thereby inhibiting its post-transcriptional expression [18, 22]. ACE2 is also post-transcriptionally regulated by miR-141-3p and miR-421 [18, 19, 23]. However, there are few studies which have investigated the expression of miR-141-3p and miR-421 in COVID-19 patients, in the present study, we aimed to investigate the serum levels of sACE2 and Ang II, as well as the expression levels of miR-141-3p and miR-421 in SARS-CoV-2-positive and negative subjects.

## Materials and methods

### Patient sample collection

One hundred eighty-eight outpatients were enrolled in the present study between June and August 2022, which divided into case and control groups. The inclusion criteria for the case group were having symptoms related to COVID-19 and a positive result of the SARS-CoV-2 quantitative real-time polymerase chain reaction (qRT‒PCR) test. For the control group, the inclusion criteria were having symptoms related to COVID-19 and a negative result of the SARS-CoV-2 qRT-PCR test.

**Fig. 1:**
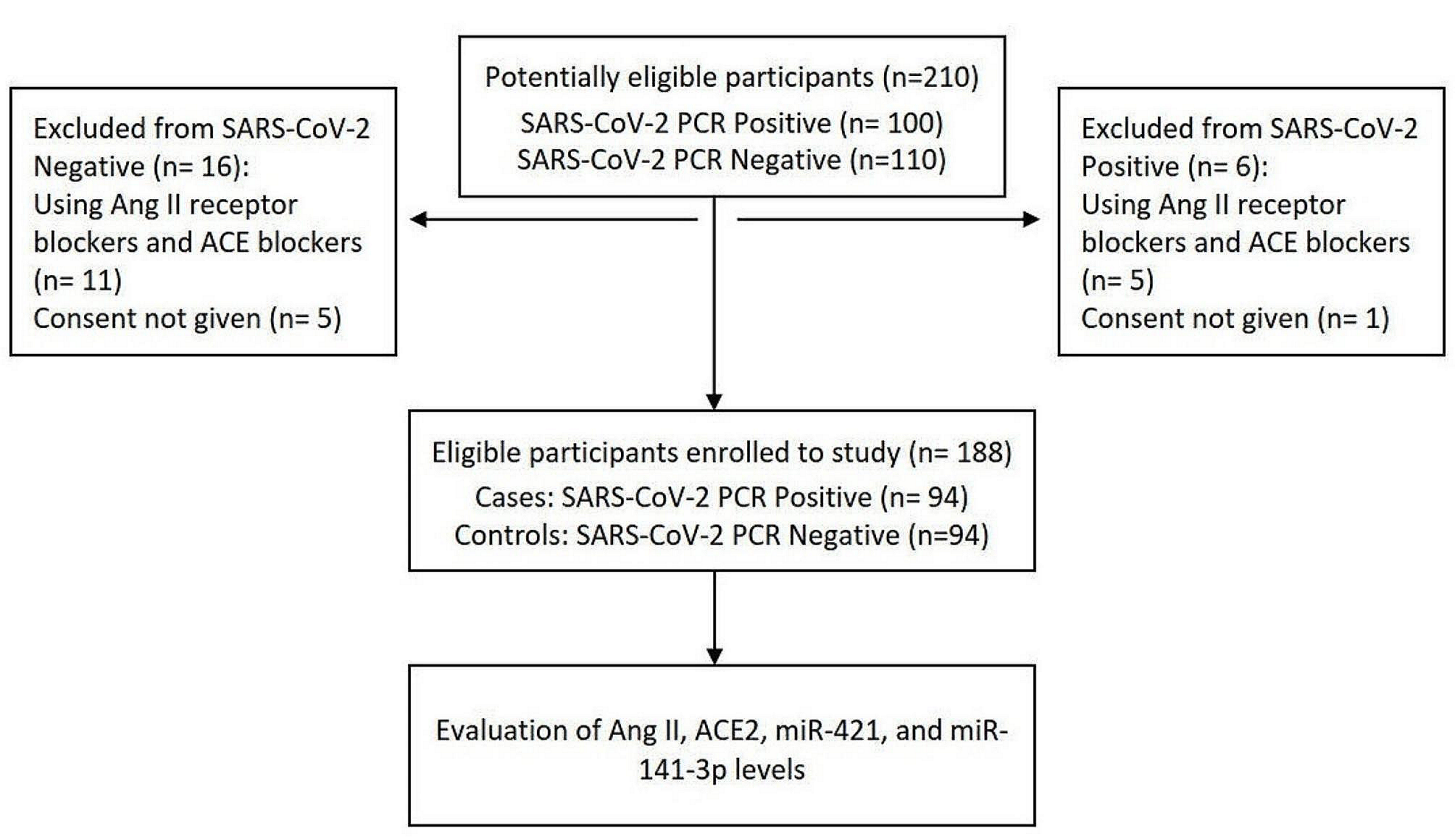
Study flow diagram

These patients were referred to a private medical laboratory for the SARS-CoV-2 PCR diagnostic test (Vahid Laboratory, Isfahan, Iran) according to the decision of their physicians. The study was approved by the medical ethics committee of Tehran University of Medical Sciences, and written informed consent was obtained from all study subjects before enrollment (ethical approval number: IR.TUMS.SPH.REC.1400.145). The swab specimens for detection of SARS-CoV-2 were obtained from both the oropharynx and nasopharynx and placed in viral transport media (VTM). Simultaneously, five milliliters of blood was obtained from all study participants in clot activator-containing tubes for the determination of Ang II, sACE2 and miRNAs levels. Participants who used any drug that might affect Ang II or ACE2 (Ang II receptor blockers and ACE blockers) or who had any kidney/heart disease were excluded. The blood samples were allowed to coagulate for 30 to 60 min at room temperature (22–25 °C) and then centrifuged at 1200 relative centrifugal force (RCF) for 15 min. The sera were separated, transferred to new tubes, and stored at -70 °C until analysis without freeze‒thaw cycles. The Fig. 1 shows the study flow diagram.

### Viral nucleic acid extraction and SARS-CoV-2 detection by qRT‒PCR

SARS-CoV-2 nucleic acid was extracted from 200 µL of each swab specimen using a commercially available viral RNA/DNA extraction kit in a silica column (Payesh Gene Rasti, Iran) according to the manufacturer’s instructions. The extraction product was stored in -70 °C until the SARS-CoV-2 detection step. SARS-CoV-2 genome detection was performed by a one-step TaqMan probe-based RT‒PCR method using a commercial kit targeting the RNA-dependent RNA polymerase (RdRp) and nucleocapsid (N) genes (Pishtaz Teb, Tehran, Iran). All RT‒PCR protocols were run based on the manufacturer’s instructions on an AusDiagnostics real-time PCR system (AusDiagnostics Pty. Ltd., Sydney, Australia).

### Analysis of serum sACE2 and Ang II levels

Serum sACE2 and Ang II levels were measured using a commercially available sandwich enzyme-linked immunosorbent assay (ELISA) kit (Bioassay Technology Laboratory, Shanghai, China) according to the manufacturer’s protocol. The assay ranges for Ang II and sACE2 were 1 to 350 ng/L and 0.05 to 20 ng/ml, respectively. The sensitivity of the assay was 0.52 ng/L for Ang II and 0.021 ng/ml for sACE2. The mean intra- and interassay coefficients of variation (CVs) for sACE2 and Ang II were < 8% and < 10%, respectively. ELISA standard curves were drawn, and the corresponding Ang II and sACE2 concentrations were subsequently determined according to the optical density (OD) data.

### Evaluation of mir-141-3p and miR-421 expression

Total RNA was extracted from all serum samples using a total RNA isolation kit (Bon Yakhteh, Tehran, Iran) according to the manufacturer’s instructions. The RNA purity and concentrations were determined using a NanoDrop ND-1000 spectrometer (Thermo Fischer Scientific, USA). Then, reverse transcription of miR-141-3p and miR-421 was carried out using the BONmiR 1st-strand cDNA synthesis kit (Bon Yakhteh, Tehran, Iran). Subsequently, qRT-PCR was performed using BON- High-Specificity microRNA qPCR Master mix (Bon Yakhteh, Tehran, Iran) and Rotor-Gene Q system (Qiagen, Hilden, Germany). The U6 snRNA was used as an internal reference gene and fold-change expression of miRNAs was evaluated using 2^−ΔΔCt^ method [24].

### Statistical analysis

The data analysis was performed using SPSS software version 18.0 (SPSS, Inc. Chicago, USA) and GraphPad Prism program 8.0 (GraphPad Software, Inc.). A nonparametric Mann‒Whitney U test was used for variables that were not normally distributed. Normality was determined by the Kolmogorov–Smirnov test. Fisher’s exact test was used to calculate the male/female ratio. In addition, the correlation between miR-141-3p and miR-421expression with sACE2 and Ang II levels was analyzed using Pearson test. *P* value < 0.05 was considered statistically significant.

## Results

### Demographic characteristics

Demographic characteristics of the patients and controls are shown in Table 1. The gender distribution in the two studied groups were not significantly different (*P* value: 0.372). Most of the patients in the case and controls were in the age group of 25–34 years.

**Table 1:** Demographic characteristics of SARS-CoV-2-positive and negative subjects

			SARS-CoV-2-positive (*N* = 94)		SARS-CoV-2-negative (*N* = 94)		Total		*P* value
	**Age, mean years** ^**a**^		35.71 ± 0.995		33.40 ± 0.932		34.56 ± 0.687		0.155
Age groups	15–24		16		7		23		0.088
	25–34		37		44		81		
	35–44		32		25		57		
	45–54		7		15		22		
	55–65		2		3		5		
	Total	94		94		188			
	**Male, N (%)**	70 (74.46%)		67 (71.27%)		137 (72.87%)		(Male/Female ratio) 0.372	
	**Female, N (%)**	24 (25.53%)		27 (28.72%)		51 (27.12%)			
symptoms	Cough	24 (25.53%)		40 (42.55%)		64 (34.04%)		0.027	
	Fever	20 (21.27%)		40 (42.55%)		60 (31.91%)			
	Gastrointestinal	23 (24.46%)		17 (18.08%)		40 (21.27%)			
	Headache	38 (40.42%)		40 (42.55%)		78 (41.48%)			
	Sore Throat	54 (57.44%)		45 (47.87%)		99 (52.65%)			

^a^ Values are presented as the mean ± SEM

### Serum sACE2 and Ang II levels

**Fig. 2:**
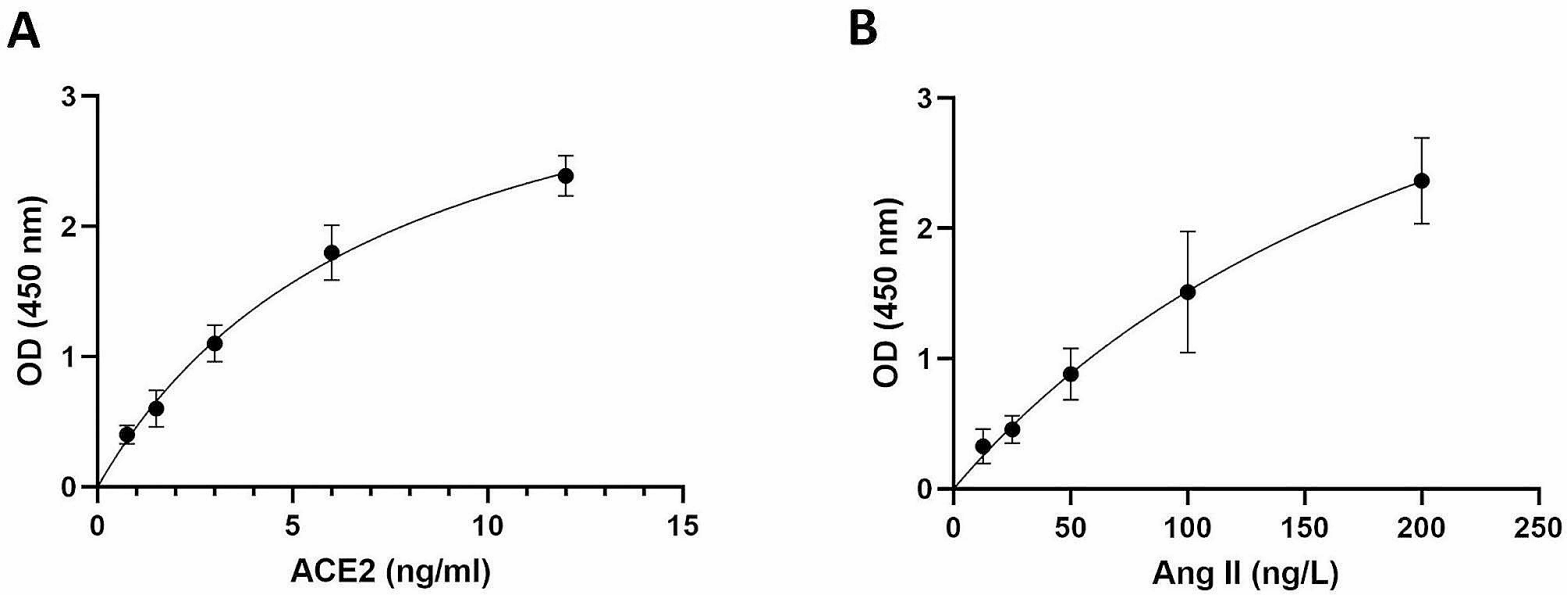
Standard curve of ELISA for sACE2 (**A**) and Ang II (**B**) quantitation. OD; optical density

Based on the ELISA protocol, standard curves were established for sACE2 and Ang II (Fig. 2A and B). Then, according to the standard curves, the serum Ang II and sACE2 levels in SARS-CoV-2 positive and negative subjects were calculated.

**Fig. 3:**
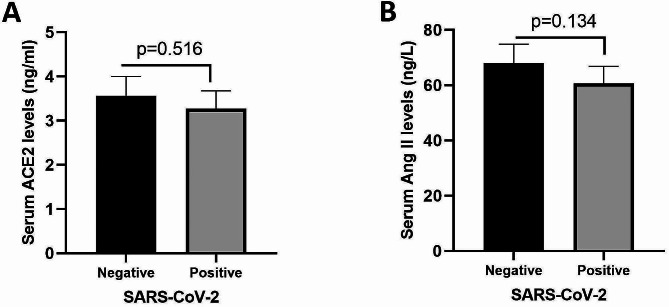
Serum sACE2 (**A**) and Ang II (**B**) levels in the SARS-CoV-2-positive (case) and -negative (control) groups. The data are presented as the mean ± SEM. The Mann-Whitney U test was used for comparisons between study groups

The mean serum sACE2 concentration in the SARS-CoV-2-positive group was 3.268 ± 0.410 ng/ml, whereas in the SARS-CoV-2 negative group, it was 3.564 ± 0.437 ng/ml. Additionally, the mean serum Ang II concentration in the SARS-CoV-2-positive and -negative groups were 60.67 ± 6.192 ng/L and 67.97 ± 6.837 ng/L, respectively. Statistical analysis revealed that there were no significant differences in the mean serum concentrations of sACE2 (*P* value: 0.516) and Ang II (*P* value: 0.134) between the SARS-CoV-2 positive and negative groups (Fig. 3A and B). Additionally, serum sACE2 and Ang II levels were analyzed according to the gender. Neither females nor males had significantly different serum levels of sACE2 (*P* value: 0.903) and Ang II (*P* value: 0.384).

### Expression of mir-141-3p and miR-421

**Fig. 4:**
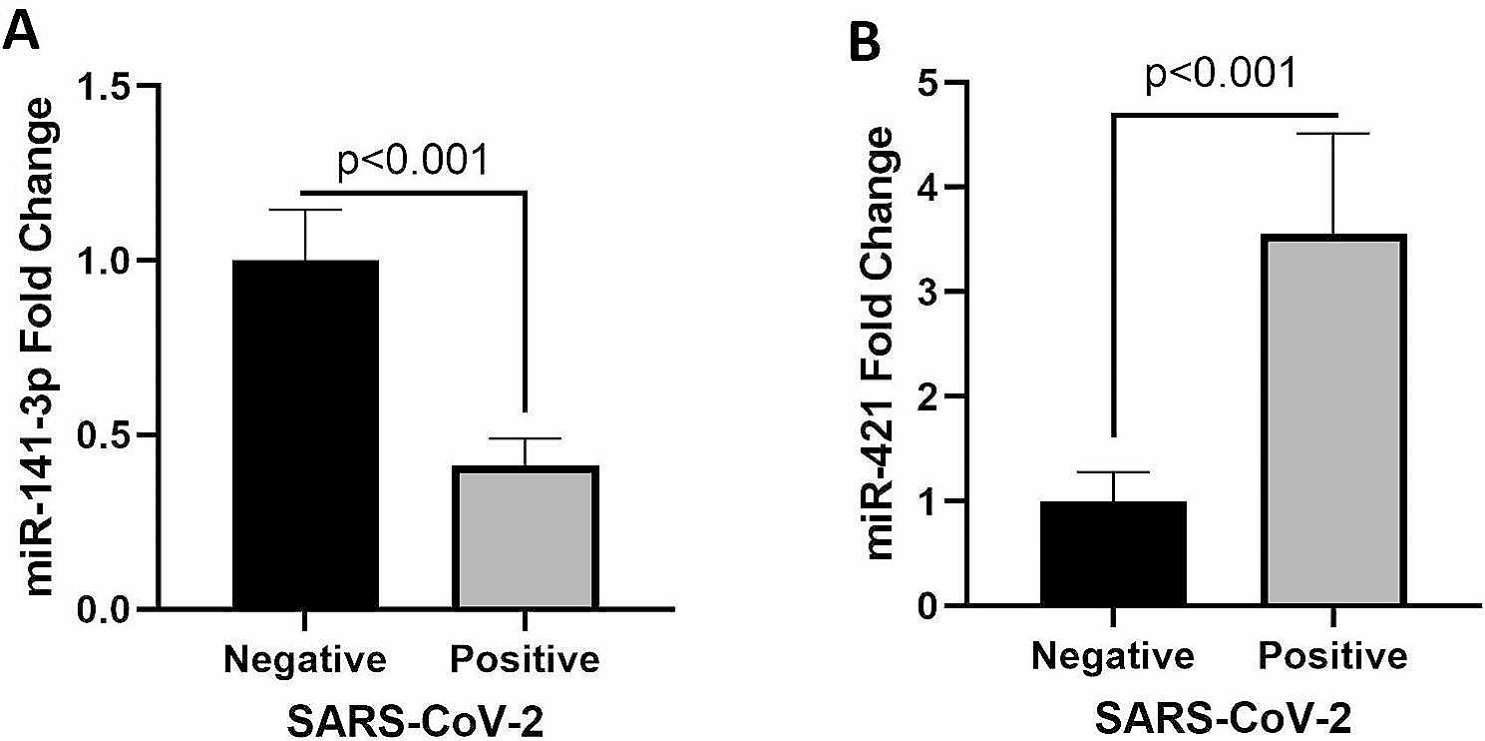
Expression of miR-141-3p (**A**) and miR-421 (**B**) levels in the SARS-CoV-2-positive (case) and -negative (control) groups. The data are presented as the mean ± SEM. The Mann-Whitney U test was used for comparisons between study groups

**Fig. 5:**
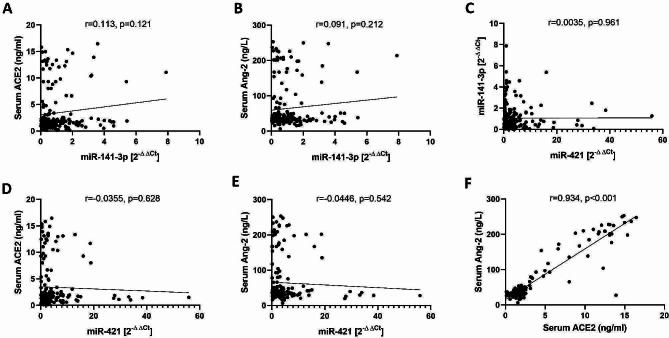
Scatter plots representing the correlation between the expression of miR-141-3p and miR-421 with sACE2 and Ang II levels in SARS-CoV-2-positive and negative subjects

The expression of miR-141-3p and miR-421 as regulators of ACE2 in the serum of SARS-CoV-2 negative and positive subjects was investigated using qRT-PCR method. Our findings indicated that the expression levels of miR-141-3p in SARS-CoV-2 positive group were significantly lower than SARS-CoV-2 negative group (*P* value < 0.001) (Fig. 4A). Meanwhile, the expression levels of miR-421 were significantly increased in the SARS-CoV-2 positive group compared to the SARS-CoV-2 negative group (*P* value < 0.001) (Fig. 4B). We next analyzed the correlations of miR-141-3p and miR-421 with sACE2 and Ang II levels in SARS-CoV-2-positive and negative population by Pearson correlation analysis. As shown in Fig. 5, no significant correlation was observed between the expression of miR-141-3p and miR-421 with the sACE2 and Ang II levels, but there was a significant positive correlation between sACE2 and Ang II levels (*P* value < 0.001).

## Discussion

After COVID-19 was declared by the World Health Organization (WHO) as a pandemic in March 2020, extensive global efforts have been made to control the pandemic and mitigate the spread of SARS-CoV-2 via mass vaccination, face mask wearing, isolation, and the use of several therapeutic agents [25, 26]. The pandemic caused by SARS-CoV-2 has highlighted the indispensable role of the RAS in the virus’s pathogenesis. The virus utilizes ACE2, an important component of RAS, as its host cell-entry receptor, and Ang II, another member of RAS, is the major biologically active component in this system. The interplay between these two elements and their potential impact on COVID-19 has been a subject of intense research [27–29]. Emerging evidence suggests that serum miRNAs, specifically miR-141-3p and miR-421, could serve as prognostic biomarkers for SARS-CoV-2 infection and may regulate sACE2 expression [19, 30]. In this study the changes in the serum levels of sACE2 and Ang II, as well as the expression level of miR-141-3p and miR-421 in SARS-CoV-2 positive and negative subjects were evaluated.

### Serum sACE2 and Ang II levels in SARS-CoV-2 positive patients compared to the controls

The results of this study showed that there was no significant differences in the mean serum concentrations of sACE2 and Ang II between the SARS-CoV-2 positive and negative groups. These results were consistent with the findings of several previous studies. Rieder et al. [31] analyzed the serum levels of sACE2 in 24 COVID-19-positive and 61 COVID-19-negative patients with similar symptoms admitted to the emergency unit. They reported no changes in serum sACE2 and Ang II levels between SARS-CoV-2 positive and the control groups. A meta-analysis study conducted by Naderi et al. found that the levels of ACE-2 were not significantly different when comparing severe COVID-19 patients with healthy controls or mild COVID-19 patients. Thus the authors proposed that sACE2 serum levels cannot be used as a biomarker to evaluate disease severity in COVID-19 patients [32]. Also, Hani et al. carried out a cross-sectional observational study on the association between serum sACE2 levels with the severity of COVID-19. They demonstrated that serum sACE2 levels did not change among SARS-CoV-2-positive and negative groups [33]. In addition, another study by Kintscher et al. reported that sACE2 levels and the Ang II/Ang I peptide ratio did not change in COVID-19 patients [34].

In contrast to these findings, several reports have shown that serum sACE2 levels were increased in COVID-19 patients and elevated sACE2 levels were associated with the severity of COVID-19 [35–39]. A study published in the scientific reports has compared the protein expression status of sACE2 in post-mortem lung specimens obtained from severe COVID-19 and non-COVID-19 patients, using immunohistochemistry (IHC). Their results showed markedly raised sACE2 protein expression in severe COVID-19 disease correlated with increased macrophage infiltration and microthrombi. The authors proposed that sACE2 might have a pathobiological role in the severity of COVID-19 [40]. In a large longitudinal 28-day study of 306 COVID-19 patients and 78 individuals without COVID-19, sACE2 plasma levels were measured, and the results showed that higher baseline sACE2 plasma levels in COVID-19 patients were significantly correlated with increased severity of the disease [41]. Increased serum sACE2 levels could be related to the spread of SARS-CoV-2 through the body and disease burden [42]. Increased lysis of mACE2-expressing cells as a result of severe infection could explain the possible causes of increased serum sACE2 levels in critically ill/deceased patients compared to mild or non-severe patients. Measurement of serum sACE2 in COVID-19 patients admitted to the hospital and control subjects showed that sACE2 serum levels were markedly greater in more severe and non-survivor cases of COVID-19 than in the milder patients [43]. Therefore, some studies have suggested that sACE2 can be used as a possible predictor marker of COVID-19 severity [33]. Contrary to these reports, several studies have demonstrated that serum/plasma sACE2 levels were significantly lower in severe COVID-19 patients than in the mild and healthy control subjects [44]. There is evidence that SARS-CoV-2 can downregulate sACE2 expression, which exacerbates disease symptoms [45, 46]. A study showed that sACE2 serum levels were significantly lower in SARS-CoV-2 patients than in SARS-CoV-2 unexposed patients and also in hospitalized SARS-CoV-2 infected patients compared to discharged SARS-CoV-2 infected patients [47]. Although many studies have been conducted on the association of serum sACE2 and COVID-19, the obtained results were controversial. There are several possible reasons for this discrepancy: (I) geographic/ancestry-related differences in sACE2 and Ang II gene expression [48, 49]. (II) disease severity, it is likely expected that if the serum levels of sACE2 and Ang II are measured in SARS-CoV-2-positive and -negative individuals, a significant difference may not be observed. However, if their levels are measured in more severe or non-survivor cases of COVID-19 compared to the milder patients, a statistically significant difference is more likely to occur. (III) The timing of the serum sample collection following a positive SARS-CoV-2 PCR test [47].

### Mir-141-3p and miR-421 expression level in SARS-CoV-2 positive patients compared to the controls

MicroRNAs, particularly those found in circulation (circulating miRNAs), have been identified as key factors in COVID-19 infection [22]. Nevertheless, there is limited understanding regarding the roles of miRNAs in COVID-19. In the present study, we evaluated the expression of miR-141-3p and miR-421 as regulators of sACE2 in the serum of SARS-CoV-2 positive and negative population. Previous studies and bioinformatics analyses have shown that miR-141-3p and miR-421 can regulate the sACE2 transcript by binding to its 3’-UTR region [19, 23]. According to Giannella et al., low serum levels of miR-141-3p, miR-4433b-5p, miR-23b-3p, miR-1-3p, and miR-155-5p were associated with increased mortality after hospitalization [50]. In a study conducted by Elemam et al. they evaluated the serum levels of sACE2, miR-421, miR-3909, miR-212-5p, and miR-4677-3p in COVID-19 patients. Their results revealed that serum levels of sACE2 and the four examined miRNAs were significantly increased in COVID-19 patients, indicating their promising role as biomarkers in COVID-19 diagnosis [18]. In line with these studies, our results also revealed that the expression level of miR-141-3p was significantly decreased in the SARS-CoV-2 positive compared to the SARS-CoV-2-negative groups. Meanwhile, the expression level of miR-421 was significantly increased in the SARS-CoV-2-positive compared to the SARS-CoV-2-negative groups.

### Limitations of the study

There were several limitations in our study. The selection of patients with mild COVID-19 infection was one of the limitations. Indeed, we did not include the factor of COVID-19 severity in our study and did not quantify serum sACE2 and Ang II in more severe and milder COVID-19 patients. Additionally, the sample size in the case and control groups was small. Moreover, we did not follow up the patients and monitor changes in serum levels of sACE2 and Ang II at different time points. However, one of the strengths of our study was the selection of the control group as SARS-CoV-2-negative subjects with some symptoms similar to those of COVID-19 patients rather than healthy individuals without symptoms.

## Conclusion

Taken together, the results of this study showed that the serum levels of sACE2 and Ang II in SARS-CoV-2 positive and negative subjects were not significantly different, but the expression of miR-141-3p and miR-421 was altered in SARS-CoV-2 and may serve as biomarkers for COVID-19 diagnosis. We suggest that future studies should investigate the serum levels of sACE2 and Ang II in SARS-CoV-2-positive and negative patients, simultaneously in more severe and milder cases of COVID-19, as well as at different time points. Additionally, we suggest that further clinical investigations with a large sample size are needed to evaluate miR-141-3p and miR-421 in SARS-CoV-2 patients to confirm the biomarker roles of these miRNAs.

## Data Availability

All data generated or analyzed during this study are included in this published article. Raw data is available from the corresponding author upon request.

## References

[1] Deslandes A, Berti V, Tandjaoui-Lambotte Y, Alloui C, Carbonnelle E, Zahar J (2020). SARS-CoV-2 was already spreading in France in late December 2019. Int J Antimicrob Agents.

[2] Basavaraju SV, Patton ME, Grimm K, Rasheed MAU, Lester S, Mills L (2020). Serologic Testing of US blood donations to identify severe Acute Respiratory Syndrome Coronavirus 2 (SARS-CoV-2)–Reactive antibodies: December 2019–January 2020. Clin Infect Dis.

[3] Merad M, Blish CA, Sallusto F, Iwasaki A. The immunology and immunopathology of COVID-19. Science (New York, NY). 2022;375(6585):1122-7. 10.1126/science.abm8108 PMC12828912 35271343

[4] Channappanavar R, Fehr AR, Vijay R, Mack M, Zhao J, Meyerholz DK (2016). Dysregulated type I interferon and inflammatory monocyte-macrophage responses cause lethal pneumonia in SARS-CoV-infected mice. Cell Host Microbe.

[5] Shatizadeh Malekshahi S, Yavarian J, Shafiei-Jandaghi NZ (2022). Usage of peptidases by SARS-CoV-2 and several human coronaviruses as receptors: a mysterious story. Biotechnol Appl Chem.

[6] Wang J, Zhao H, An Y (2022). ACE2 shedding and the role in COVID-19. Front Cell Infect Microbiol.

[7] Beyerstedt S, Casaro EB, Rangel ÉB (2021). COVID-19: angiotensin-converting enzyme 2 (ACE2) expression and tissue susceptibility to SARS-CoV-2 infection. Eur J Clin Microbiol Infect Diseases: Official Publication Eur Soc Clin Microbiol.

[8] Shirbhate E, Pandey J, Patel VK, Kamal M, Jawaid T, Gorain B (2021). Understanding the role of ACE-2 receptor in pathogenesis of COVID-19 disease: a potential approach for therapeutic intervention. Pharmacol Rep.

[9] Reindl-Schwaighofer R, Hödlmoser S, Domenig O, Krenn K, Eskandary F, Krenn S (2022). The systemic renin-angiotensin system in COVID-19. Sci Rep.

[10] Bourgonje AR, Abdulle AE, Timens W, Hillebrands JL, Navis GJ, Gordijn SJ (2020). Angiotensin-converting enzyme 2 (ACE2), SARS‐CoV‐2 and the pathophysiology of coronavirus disease 2019 (COVID‐19). J Pathol.

[11] Imai Y, Kuba K, Penninger JM (2006). The renin–angiotensin system in acute respiratory distress syndrome. Drug Discovery Today: Disease Mech.

[12] Vrigkou E, Tsangaris I, Bonovas S, Tsantes A, Kopterides P (2017). The evolving role of the renin–angiotensin system in ARDS. Crit Care.

[13] Laghlam D, Jozwiak M, Nguyen LS (2021). Renin–angiotensin–aldosterone system and immunomodulation: a state-of-the-art review. Cells.

[14] Warner FJ, Lew RA, Smith AI, Lambert DW, Hooper NM, Turner AJ (2005). Angiotensin-converting enzyme 2 (ACE2), but not ACE, is preferentially localized to the apical surface of polarized kidney cells. J Biol Chem.

[15] Samavati L, Uhal BD. ACE2, much more than just a receptor for SARS-COV-2. Front Cell Infect Microbiol. 2020:317. 10.3389/fcimb.2020.00317 PMC7294848 32582574

[16] Belouzard S, Chu VC, Whittaker GR. Activation of the SARS coronavirus spike protein via sequential proteolytic cleavage at two distinct sites. Proceedings of the National Academy of Sciences. 2009;106(14):5871-6. 10.1073/pnas.0809524106 PMC2660061 19321428

[17] Huang F, Guo J, Zou Z, Liu J, Cao B, Zhang S (2014). Angiotensin II plasma levels are linked to disease severity and predict fatal outcomes in H7N9-infected patients. Nat Commun.

[18] Elemam NM, Hasswan H, Aljaibeji H, Sharif-Askari NS, Halwani R, Taneera J et al. Profiling levels of serum microRNAs and Soluble ACE2 in COVID-19 patients. Life (Basel). 2022;12(4). 10.3390/life12040575 PMC9027848 35455065

[19] Nersisyan S, Shkurnikov M, Turchinovich A, Knyazev E, Tonevitsky A (2020). Integrative analysis of miRNA and mRNA sequencing data reveals potential regulatory mechanisms of ACE2 and TMPRSS2. PLoS ONE.

[20] Sodagar H, Khadem Ansari MH, Asghari R, Alipour S (2022). Evaluation of serum levels of MicroRNA-200 C and ACE2 gene expression in severe and mild phases of patients with COVID-19. Iran J Allergy Asthma Immunol.

[21] Mirzavi F, Ebrahimi S, Ghazvini K, Hasanian SM, Hashemy SI (2019). Diagnostic, Prognostic, and therapeutic potencies of circulating miRNAs in Acute myocardial infarction. Crit Rev Eukaryot Gene Expr.

[22] Jankovic M, Nikolic D, Novakovic I, Petrovic B, Lackovic M, Santric-Milicevic M. miRNAs as a potential biomarker in the COVID-19 infection and complications Course, Severity, and Outcome. Diagnostics (Basel). 2023;13(6). 10.3390/diagnostics13061091 PMC10047241 36980399

[23] Lambert DW, Lambert LA, Clarke NE, Hooper NM, Porter KE, Turner AJ (2014). Angiotensin-converting enzyme 2 is subject to post-transcriptional regulation by miR-421. Clin Sci (Lond).

[24] Livak KJ, Schmittgen TD (2001). Analysis of relative gene expression data using real-time quantitative PCR and the 2(-Delta Delta C(T)) method. Methods.

[25] Haug N, Geyrhofer L, Londei A, Dervic E, Desvars-Larrive A, Loreto V (2020). Ranking the effectiveness of worldwide COVID-19 government interventions. Nat Hum Behav.

[26] Güner HR, Hasanoğlu İ, Aktaş F (2020). COVID-19: Prevention and control measures in community. Turk J Med Sci.

[27] Xavier LL, Neves PFR, Paz LV, Neves LT, Bagatini PB, Timmers LFSM (2021). Does angiotensin II peak in response to SARS-CoV-2?. Front Immunol.

[28] Babajani F, Kakavand A, Mohammadi H, Sharifi A, Zakeri S, Asadi S (2021). COVID-19 and renin angiotensin aldosterone system: Pathogenesis and therapy. Health Sci Rep.

[29] Faraji SN, Raee MJ, Hashemi SMA, Daryabor G, Tabrizi R, Dashti FS (2022). Human interaction targets of SARS-CoV-2 spike protein: a systematic review. Eur J Inflamm.

[30] Elemam NM, Hasswan H, Aljaibeji H, Sharif-Askari NS, Halwani R, Taneera J (2022). Profiling levels of serum microRNAs and soluble ACE2 in COVID-19 patients. Life.

[31] Rieder M, Wirth L, Pollmeier L, Jeserich M, Goller I, Baldus N (2021). Serum ACE2, angiotensin II, and aldosterone levels are unchanged in patients with COVID-19. Am J Hypertens.

[32] Naderi N, Rahimzadeh M (2023). The role of Soluble ACE2 as a prognostic marker in severe COVID-19: a brief Meta-analysis. Endocrine, metabolic & Immune disorders-drug targets (formerly current drug targets-Immune. Endocr Metabolic Disorders).

[33] Bani Hani A, Abu Tarboush N, Ma BA, Alabhoul F, Alansari F, Abuhani A (2022). Serum ACE2 level is Associated with severe SARS-CoV-2 infection: a cross-sectional observational study. Biomark Insights.

[34] Kintscher U, Slagman A, Domenig O, Röhle R, Konietschke F, Poglitsch M (2020). Plasma angiotensin peptide profiling and ACE (angiotensin-converting enzyme)-2 activity in COVID-19 patients treated with pharmacological blockers of the renin-angiotensin system. Hypertension.

[35] Nagy B, Fejes Z, Szentkereszty Z, Sütő R, Várkonyi I, Ajzner É (2021). A dramatic rise in serum ACE2 activity in a critically ill COVID-19 patient. Int J Infect Dis.

[36] van Lier D, Kox M, Santos K, van der Hoeven H, Pillay J, Pickkers P. Increased blood angiotensin converting enzyme 2 activity in critically ill COVID-19 patients. ERJ Open Res. 2021;7(1). 10.1183/23120541.00848-2020 PMC7848790 33738305

[37] Lundström A, Ziegler L, Havervall S, Rudberg AS, Von Meijenfeldt F, Lisman T (2021). Soluble angiotensin-converting enzyme 2 is transiently elevated in COVID‐19 and correlates with specific inflammatory and endothelial markers. J Med Virol.

[38] Shevchuk O, Pak A, Palii S, Ivankiv Y, Kozak K, Korda M (2023). Blood ACE2 protein level correlates with COVID-19 severity. Int J Mol Sci.

[39] Wissing SI, Obeid R, Rädle-Hurst T, Rohrer T, Herr C, Schöpe J (2022). Concentrations of soluble angiotensin converting enzyme 2 (sACE2) in children and adults with and without COVID-19. J Clin Med.

[40] Gheware A, Ray A, Rana D, Bajpai P, Nambirajan A, Arulselvi S (2022). ACE2 protein expression in lung tissues of severe COVID-19 infection. Sci Rep.

[41] Kragstrup TW, Singh HS, Grundberg I, Nielsen AL-L, Rivellese F, Mehta A (2021). Plasma ACE2 predicts outcome of COVID-19 in hospitalized patients. PLoS ONE.

[42] Perrotta F, Matera MG, Cazzola M, Bianco A (2020). Severe respiratory SARS-CoV2 infection: does ACE2 receptor matter?. Respir Med.

[43] Bani Hani A, Abu Tarboush N, Bani Ali M, Alabhoul F, Alansari F, Abuhani A (2022). Serum ACE2 level is Associated with severe SARS-CoV-2 infection: a cross-sectional observational study. Biomark Insights.

[44] Robertson J, Nellgård B, Hultén LM, Nilsson S, Dalla K, Börjesson M (2022). Sex difference in circulating soluble form of ACE2 protein in moderate and severe COVID-19 and healthy controls. Front Med.

[45] Triana S, Metz-Zumaran C, Ramirez C, Kee C, Doldan P, Shahraz M (2021). Single‐cell analyses reveal SARS‐CoV‐2 interference with intrinsic immune response in the human gut. Mol Syst Biol.

[46] Seltzer S (2020). Linking ACE2 and angiotensin II to pulmonary immunovascular dysregulation in SARS-CoV-2 infection. Int J Infect Dis.

[47] Díaz-Troyano N, Gabriel-Medina P, Weber S, Klammer M, Barquín-DelPino R, Castillo-Ribelles L (2022). Soluble angiotensin-converting enzyme 2 as a prognostic biomarker for disease progression in patients infected with SARS-CoV-2. Diagnostics.

[48] Maza MDC, Úbeda M, Delgado P, Horndler L, Llamas MA, van Santen HM (2022). ACE2 serum levels as Predictor of Infectability and Outcome in COVID-19. Front Immunol.

[49] Narula S, Yusuf S, Chong M, Ramasundarahettige C, Rangarajan S, Bangdiwala SI (2020). Plasma ACE2 and risk of death or cardiometabolic diseases: a case-cohort analysis. Lancet.

[50] Giannella A, Riccetti S, Sinigaglia A, Piubelli C, Razzaboni E, Di Battista P (2022). Circulating microRNA signatures associated with disease severity and outcome in COVID-19 patients. Front Immunol.

